# Fucoxanthin Induces Ferroptosis in Cancer Cells via Downregulation of the Nrf2/HO−1/GPX4 Pathway

**DOI:** 10.3390/molecules29122832

**Published:** 2024-06-14

**Authors:** Hao-Fei Du, Jia-Wei Wu, Yu-Shan Zhu, Zheng-Hao Hua, Si-Zhou Jin, Jin-Chao Ji, Cai-Sheng Wang, Guo-Ying Qian, Xu-Dong Jin, Hao-Miao Ding

**Affiliations:** Hwamei College of Life and Health Sciences, Zhejiang Wanli University, Ningbo 315100, China; youzanzizwu@163.com (H.-F.D.); wujiawei29@163.com (J.-W.W.); zhuyszwu@163.com (Y.-S.Z.); huazh0214@163.com (Z.-H.H.); jsz2023010120@163.com (S.-Z.J.); jijinchaozwu@163.com (J.-C.J.); wangcs0528@163.com (C.-S.W.); qiangy@zwu.edu.cn (G.-Y.Q.)

**Keywords:** fucoxanthin, anti−cancer, ferroptosis, Nrf2/HO−1/GPX4 pathway

## Abstract

This study investigated the mechanism by which fucoxanthin acts as a novel ferroptosis inducer to inhibit tongue cancer. The MTT assay was used to detect the inhibitory effects of fucoxanthin on SCC−25 human tongue squamous carcinoma cells. The levels of reactive oxygen species (ROS), mitochondrial membrane potential (MMP), glutathione (GSH), superoxide dismutase (SOD), malondialdehyde (MDA), and total iron were measured. Reverse transcription–quantitative polymerase chain reaction (RT−qPCR) and Western blotting were used to assess glutathione peroxidase 4 (GPX4), nuclear factor erythroid 2−related factor 2 (Nrf2), Keap1, solute carrier family 7 member 11 (SLC7A11), transferrin receptor protein 1 (TFR1), p53, and heme oxygenase 1 (HO−1) expression. Molecular docking was performed to validate interactions. Compared with the control group, the activity of fucoxanthin−treated SCC−25 cells significantly decreased in a dose− and time−dependent manner. The levels of MMP, GSH, and SOD significantly decreased in fucoxanthin−treated SCC−25 cells; the levels of ROS, MDA, and total iron significantly increased. mRNA and protein expression levels of Keap1, GPX4, Nrf2, and HO−1 in fucoxanthin−treated cells were significantly decreased, whereas levels of TFR1 and p53 were significantly increased, in a concentration−dependent manner. Molecular docking analysis revealed that binding free energies of fucoxanthin with p53, SLC7A11, GPX4, Nrf2, Keap1, HO−1, and TFR1 were below −5 kcal/mol, primarily based on active site hydrogen bonding. Our findings suggest that fucoxanthin can induce ferroptosis in SCC−25 cells, highlighting its potential as a treatment for tongue cancer.

## 1. Introduction

Tongue cancer is a malignant oral tumor that mostly occurs in adults aged 40–60 years [1]. Its complex etiology is associated with foreign body stimulation, radiation injury, immunodeficiency, poor oral hygiene, and other factors. The tongue’s abundant blood vessels and lymphatic tissue, as well as its frequent mechanical movement, lead to early hematogenous and lymph node metastasis; it may reach the lung in later stages [2]. Current tongue cancer treatment mainly involves surgical resection of the primary lesion combined with neck dissection, which is partially effective [3]. However, postoperative recurrence and metastasis are common, and patient prognosis is poor. Therefore, to improve treatment outcomes, improve quality of life, and reduce the rates of recurrence and metastasis, novel drugs with good efficacy and few side effects have become a focus of clinical studies. Traditional Chinese medicine has received considerable attention in tongue cancer research, based on its long history. Traditional Chinese medicine has many advantages, such as low toxicity and few side effects, minimal drug resistance, and synergy with various chemotherapy drugs.

**Figure 1:**
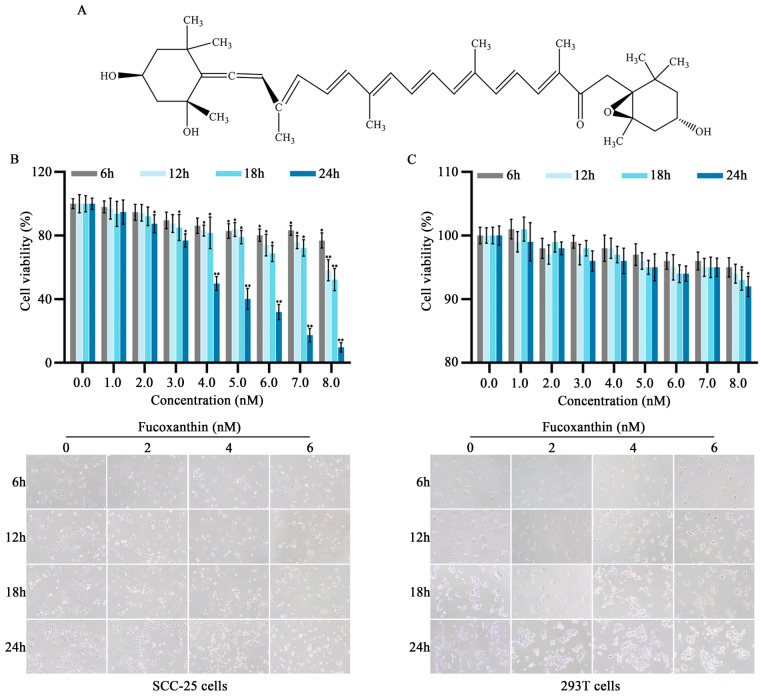
Effects of different fucoxanthin concentrations (0, 1, 2, 3, 4, 5, 6, 7, or 8 nM) for 6, 12, 18, or 24 h on SCC−25 tongue cancer and 293T embryonic kidney cell viability. (**A**) Chemical structure of fucoxanthin. (**B**) SCC−25 cell viability after fucoxanthin treatment relative to control group (no drug treatment). (**C**) 293T cell viability after fucoxanthin treatment relative to control group. Data are means ± standard deviations (SDs; *n* = 5). * *p* < 0.05, ** *p* < 0.01, compared with control.

Fucoxanthin is an oxygenic carotenoid that widely exists in algae, marine phytoplankton, aquatic shellfish, and invertebrates [4]. Its molecular formula is C_42_H_58_O_6_, molecular mass is 658.91, and chemical structure is shown in Figure 1A. Studies have demonstrated fucoxanthin’s anti−angiogenic, anti−tumor, anti−inflammatory, anti−oxidative, anti−Alzheimer’s, and neuroprotective effects [5,6]. Fucoxanthin inhibits tumor cell proliferation by inhibiting the cell cycle in the G0/G1 or G1 phase [7]. The molecular mechanism may involve upregulation of cyclin−dependent kinase inhibitor protein expression and downregulation of one or more cyclin−dependent kinases [8]. Additionally, recent research indicated that fucoxanthin inhibits tongue cancer CAL−27 cell migration and invasion by inhibiting the expression of protein kinase B (AKT)/mechanistic target of rapamycin (mTOR) pathway components [9]. Similarly, fucoxanthin promotes apoptosis and autophagy in mouse cancer cells by reducing the phosphorylation of Akt, its downstream proteins, and apoptosis−related proteins; it also reduces DNA damage in normal cells and tumor cell metastasis and invasion by inhibiting chronic inflammation [10,11,12]. These findings demonstrated that fucoxanthin has potential as an anti−tumor agent with inhibitory effects on tumor cell proliferation, migration, and invasion. However, its anti−cancer mechanism within tongue cancer is not fully understood.

Ferroptosis is a recently discovered form of regulated cell death [13]. There is increasing evidence that ferroptosis plays an important role in regulating the occurrence and progression of various diseases. For example, hepatocytes exhibit key characteristics of ferroptosis during acute liver injury [14]. The morphological appearance of ferroptosis comprises shrunken mitochondria, increased mitochondrial membrane density, reduced or absent mitochondrial cristae, and rupture of the outer mitochondrial membrane. Factors affecting ferroptosis include ferrous iron, reactive oxygen species (ROS), and glutathione peroxidase 4 (GPX4) [15]. Iron metabolism plays a vital role in ferroptosis, and iron homeostasis is closely associated with cellular sensitivity to ferroptosis [16]. Iron absorbed from the external environment is bound to transferrin (TF) in plasma and transported to various parts of the body; therefore, TF is crucial for regulating ferroptosis [17]. Intracellular iron enters mitochondria through solute carriers in the mitochondrial inner membrane (e.g., SLC25A37 and SLC25A22) and participates in the biosynthesis of iron−sulfur clusters (ISCs) and heme. ISCs regulate intracellular iron homeostasis and avoid iron overload−mediated ROS production [18]. This study investigated the effects of fucoxanthin on ferroptosis within SCC−25 human tongue squamous carcinoma cells in vitro; it sought to provide a theoretical and experimental basis for clinical applications of fucoxanthin in tongue cancer treatment.

## 2. Results

### 2.1. Inhibitory Effects of Fucoxanthin on SCC−25 Tongue Cancer Cells

To evaluate the in vitro anti−cancer effects of fucoxanthin on tongue cancer, SCC−25 cells were treated with various mass concentrations of fucoxanthin extract for 6, 12, 18, and 24 h, as shown in Figure 1B. Additionally, the 293T cell line (i.e., normal human embryonic kidney cells) was utilized to analyze fucoxanthin cytotoxicity (Figure 1C). At concentrations exceeding 7 nM, fucoxanthin exhibited inhibitory effects on both SCC−25 and normal cells, although the effects on normal cells were substantially weaker. Compared with the control group, the rate of SCC−25 cell survival decreased as fucoxanthin dose and exposure duration increased (*p* < 0.05 and *p* < 0.01, respectively). The IC_50_ values for fucoxanthin in SCC−25 cells at 6, 12, 18, and 24 h were 31.56, 12.93, 10.36, and 4.17 nM, respectively. To exclude the effects of nonspecific cytotoxicity, 2, 4, and 6 nM fucoxanthin were used as the low, medium, and high doses of fucoxanthin in subsequent experiments.

### 2.2. Effects of Fucoxanthin on ROS in SCC−25 Cells

**Figure 2:**
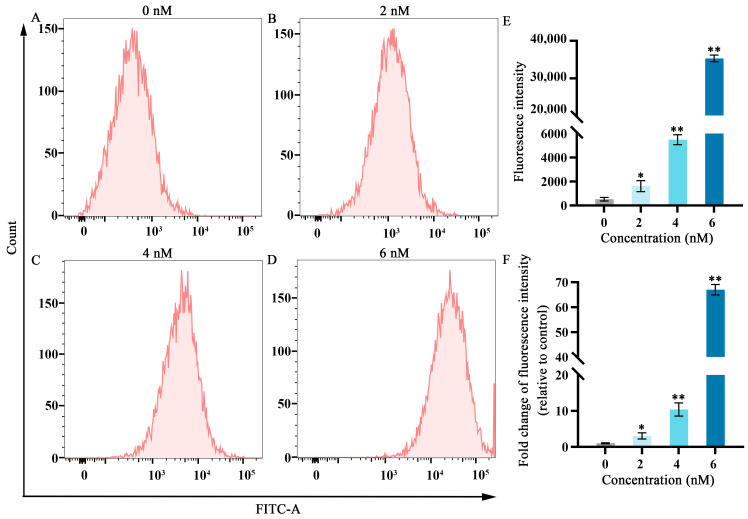
Effects of different fucoxanthin concentrations (0, 2, 4, or 6 nM) for 24 h on ROS production in SCC−25 cells. (**A**) Control group, (**B**) 2 nM fucoxanthin, (**C**) 4 nM fucoxanthin, (**D**) 6 nM fucoxanthin, and (**E**) quantitative analysis of fluorescence intensity in SCC−25 cells. (**F**) Fold change of fluorescence intensity (relative to control) in SCC−25 cells. * *p* < 0.05, ** *p* < 0.01, compared with control.

Elevated ROS levels represent an important feature of ferroptosis [19]. As shown in Figure 2, after treatment with fucoxanthin for 24 h, ROS levels significantly increased compared with the control group in a dose−dependent manner. At fucoxanthin concentrations of 6 nM, ROS production increased by almost 60−fold.

### 2.3. Effects of Fucoxanthin on Malondialdehyde (MDA), Superoxide Dismutase (SOD), Glutathione (GSH), and Iron (Fe) in SCC−25 Cells

**Figure 3:**
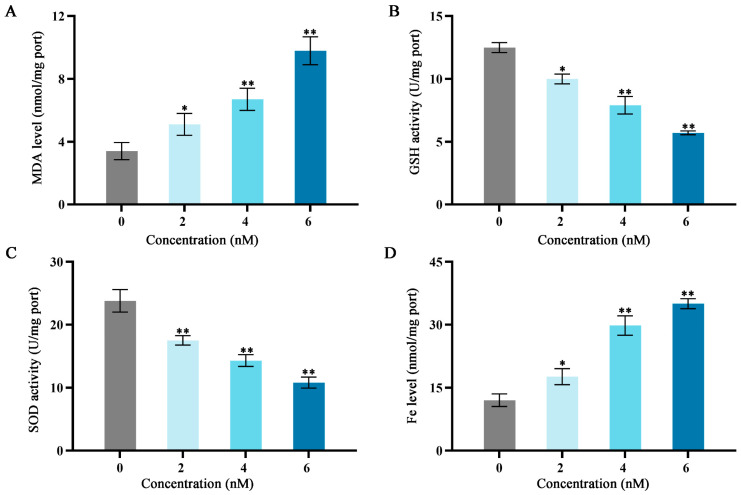
Effects of different fucoxanthin concentration (0, 2, 4, or 6 nM) for 24 h on antioxidant enzyme activities, Fe level, and MDA level in SCC−25 cells. (**A**) MDA level, (**B**) GSH activity, (**C**) SOD activity, and (**D**) Fe level. * *p* < 0.05, ** *p* < 0.01, compared with control.

As shown in Figure 3, after treatment with fucoxanthin for 24 h, compared with the control group, the levels of MDA and Fe increased as the fucoxanthin concentration increased; the middle and high dose groups exhibited statistically significant differences relative to the control group (*p* < 0.01; Figure 3A,D). The GSH and SOD activities substantially decreased as the fucoxanthin concentration increased; the middle and high dose groups displayed statistically significant differences relative to the control group (*p* < 0.01; Figure 3B,C).

### 2.4. Effects of Fucoxanthin on Apoptosis in SCC−25 Cells

**Figure 4:**
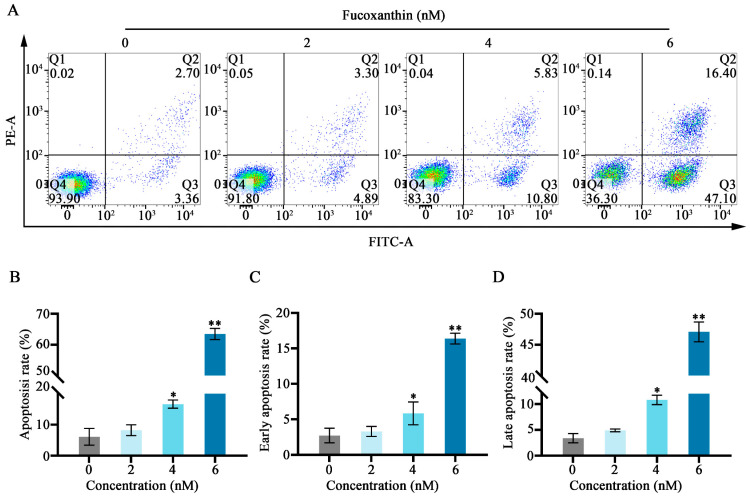
Fucoxanthin−induced apoptosis in SCC−25 cells. Data are means ± SDs (*n* = 3). (**A**) Flow cytometry was utilized to evaluate apoptosis after 24 h of treatment with 0, 2, 4, and 6 nM of fucoxanthin. (**B**–**D**) Graphs showing the effects of fucoxanthin on the overall, early, and late apoptosis rates in SCC−25 cells. * *p* < 0.05, ** *p* < 0.01 compared with the control group.

Annexin−V/propidium iodide (PI) staining and flow cytometry were used to detect the effects of fucoxanthin for 24 h on apoptosis in SCC−25 cells. In the control group, the apoptosis rate was 6.06%. Treatment with 2 nM fucoxanthin increased the rate to 8.91%; treatment with 4 nM and 6 nM further increased the rate to 16.63% and 63.50%, respectively. As the fucoxanthin concentration increased, the rates of apoptosis, early apoptosis, and late apoptosis in SCC−25 cells increased (Figure 4B,C). Thus, fucoxanthin effectively promoted early apoptosis in SCC−25 cells; its effect on late apoptosis was less pronounced. 

### 2.5. Effects of Fucoxanthin on Mitochondrial Membrane Potential in SCC−25 Tongue Cancer Cells

**Figure 5:**
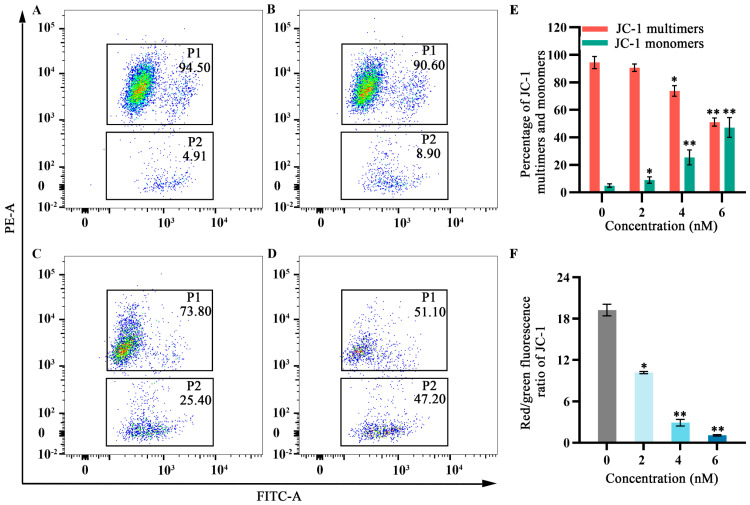
Effects of fucoxanthin for 24 h on mitochondrial membrane potential in SCC−25 cells. Data are means ± SDs (*n* = 3). (**A**) Control group, (**B**) 2 nM fucoxanthin, (**C**) 4 nM fucoxanthin, (**D**) 6 nM fucoxanthin, (**E**) percentages of JC−1 multimers and monomers, and (**F**) JC−1 red/green fluorescence ratio. * *p* < 0.05, ** *p* < 0.01, compared with control.

As cellular energy factories, mitochondria maintain high membrane potential; disruption of this potential can lead to apoptosis. Flow cytometry was used to observe the effects of fucoxanthin for 24 h on mitochondrial membrane potential in SCC−25 cells. In the control group, the JC−1 monomer proportion in SCC−25 cells was 4.91%. Treatment with 2 nM fucoxanthin increased the proportion to 8.90%; treatment with 4 nM and 6 nM further increased the proportion to 25.40% and 47.20%, respectively (Figure 5A–D). Low mitochondrial membrane potential prevented JC−1 accumulation in the mitochondrial matrix; this lack of accumulation resulted in green fluorescence. High mitochondrial membrane potential enabled JC−1 to accumulate in the mitochondrial matrix and form polymers, thereby producing red fluorescence. JC−1 red fluorescence intensity significantly decreased after fucoxanthin treatment, whereas green fluorescence intensity significantly increased (*p* < 0.05 and *p* < 0.01, respectively; Figure 5E,F), indicating that fucoxanthin substantially disrupted the mitochondrial membrane potential in SCC−25 cells. These results suggest that fucoxanthin can influence mitochondrial function in tongue cancer.

### 2.6. Effects of Fucoxanthin on mRNA Expression Levels of p53, SLC7A11, GPX4, Nuclear Factor Erythroid 2−Related Factor 2 (Nrf2), Keap1, Heme Oxygenase 1 (HO−1), and TF Receptor 1 (TFR1) in SCC−25 Tongue Cancer Cells

**Figure 6:**
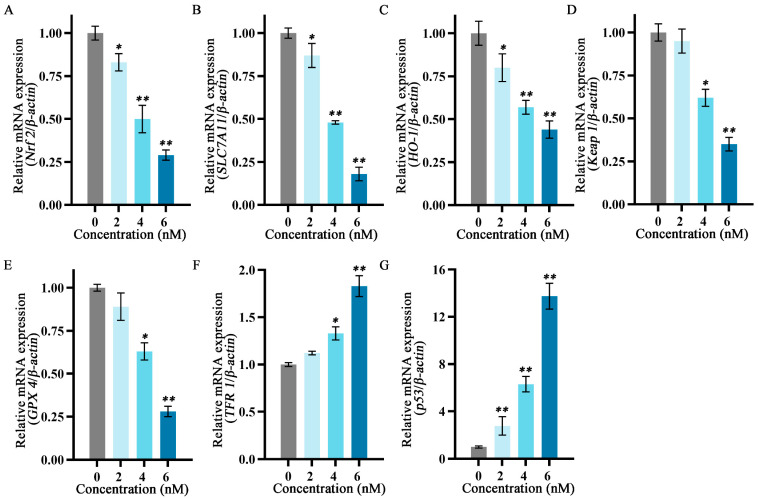
Effects of different fucoxanthin concentrations (0, 2, 4, or 6 nM) for 24 h on ferroptosis−related gene expression in SCC−25 cells. Data are means ± SDs (*n* = 3). (**A**–**G**) Quantitative measurements of the effects of fucoxanthin on *Nrf2*, *SLC7A11*, *HO−1*, *Keap1*, *GPX4*, *TFR1*, and *p53* mRNA expression in SCC−25 cells, respectively. * *p* < 0.05, ** *p* < 0.01 compared with the control group.

To explore the relationship between the inhibitory effects of fucoxanthin for 24 h on SCC−25 cells and the onset of ferroptosis, the mRNA expression levels of ferroptosis−related factors *p53*, *SLC7A11*, *GPX4*, *Nrf2*, *Keap1*, *HO−1*, and *TFR1* were measured. The relative expression levels of *Nrf2*, *HO−1*, *SLC7A11*, *Keap1*, and *GPX4* gradually decreased as the fucoxanthin concentration increased (Figure 6A–E). Treatment with at least 4 nM fucoxanthin significantly reduced the expression levels of *Keap1* and *GPX4* (*p* < 0.05). At fucoxanthin concentrations of ≥4 nM and ≥2 nM, the expression levels of *TFR1* and *p53* were significantly increased (*p* < 0.05 and *p* < 0.01, respectively).

### 2.7. Effects of Fucoxanthin on Protein Expression Levels of p53, SLC7A11, GPX4, Nrf2, Keap1, HO−1, and TFR1 in SCC−25 Tongue Cancer Cells

**Figure 7:**
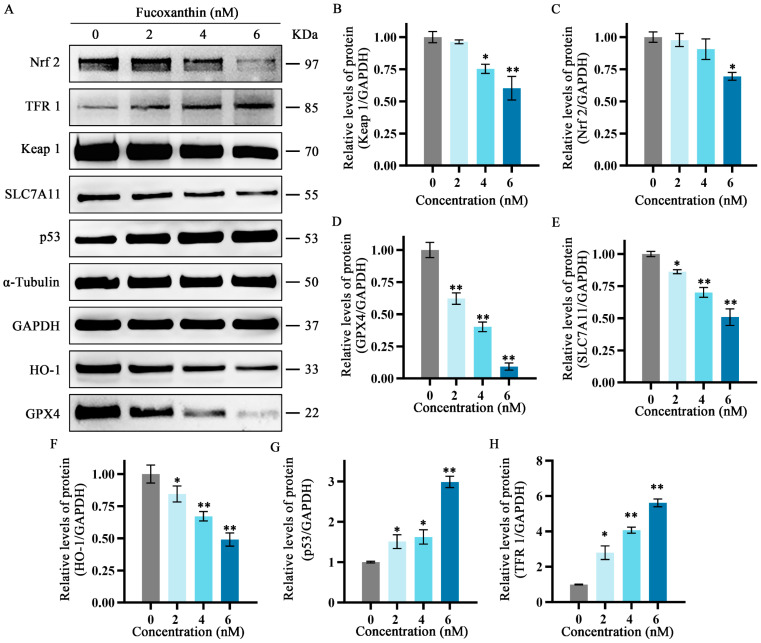
Effects of different fucoxanthin concentrations (0, 2, 4, or 6 nM) for 24 h on the Keap1/Nrf2/HO−1 signaling pathway in SCC−25 cells. Data are means ± SDs (*n* = 3). (**A**) Qualitative depiction of the effects of fucoxanthin on ferroptosis−related proteins in SCC−25 cells. (**B**–**H**) Quantitative measurements of the effects of fucoxanthin on Keap1, Nrf2, p53, SLC7A11, HO−1, GPX4, and TFR1 protein expression in SCC−25 cells, respectively. * *p* < 0.05, ** *p* < 0.01, compared with control.

To further explore the relationship between the inhibitory effects of fucoxanthin for 24 h on SCC−25 cells and the onset of ferroptosis, the protein expression levels of ferroptosis−related factors p53, SLC7A11, GPX4, Nrf2, Keap1, HO−1, and TFR1 were measured. The relative expression levels of Nrf2, HO−1, SLC7A11, Keap1, and GPX4 gradually decreased as the fucoxanthin concentration increased (Figure 7A–E). Treatment with at least 2 nM fucoxanthin significantly reduced the expression levels of Keap 1, HO−1, and SLC7A11 (*p* < 0.05). Treatment with at least 4 nM fucoxanthin significantly reduced the expression levels of Keap1 and Nrf2 (*p* < 0.05). At fucoxanthin concentrations of ≥4 nM and ≥2 nM, the expression levels of TFR1 and p53 were significantly increased (*p* < 0.01 and *p* < 0.05, respectively).

### 2.8. Molecular Docking Validation

**Figure 8:**
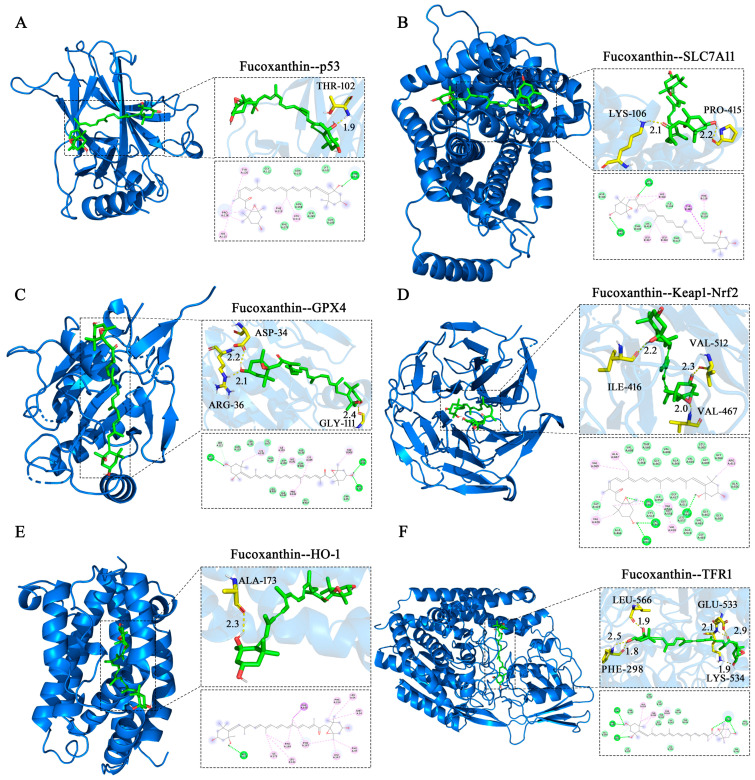
Molecular docking of fucoxanthin with target proteins. Optimal conformations of fucoxanthin binding to p53 (**A**), SLC7A11 (**B**), GPX4 (**C**), Keap1–Nrf2 (**D**), HO−1 (**E**), and TFR1 (**F**), with key residues around each binding site. Hydrogen bonds are presented as yellow dashed lines with distances in angstroms (Å). Two−dimensional schematic diagrams depict interactions of fucoxanthin with active sites of target proteins. Dotted lines represent hydrogen bonds (dark green), π–σ T−shaped forces (dark purple), and hydrophobic interactions (purple) with surrounding amino acid residues. Acidic residues in light green denote van der Waals forces.

Molecular docking simulations were performed to investigate interactions between fucoxanthin and its target proteins (p53, SLC7A11, GPX4, Keap1, HO−1, and TFR1). PyMOL 2.5.2 software was used to visualize fucoxanthin–target protein complexes, obtaining binding models that clearly revealed interacting amino acid residues at binding sites. Fucoxanthin formed hydrogen bonds with THR−102 (1.9 Å) on the p53 protein, with a calculated binding free energy of −7.3 kcal/mol (Figure 8A). Fucoxanthin also formed hydrogen bonds with LYS−106 (2.1 Å) and PRO−415 (2.2 Å) on the SLC7A11 protein; the calculated binding free energy was −5.81 kcal/mol (Figure 8B). Concerning the GPX4 protein, fucoxanthin formed hydrogen bonds with the ARG−36 (2.1 Å), GLY−111 (2.4 Å), and ASP−34 (2.2 Å) residues, with a calculated binding free energy of −7.23 kcal/mol (Figure 8C). Additionally, fucoxanthin formed hydrogen bonds with ILE−416 (2.2 Å), VAL−512 (2.3 Å), and VAL−467 (2.0 Å) on the Keap1–Nrf2 protein; the calculated binding free energy was −11.26 kcal/mol (Figure 8D). Fucoxanthin formed hydrogen bonds with ALA−173 (2.3 Å) on the HO−1 protein, with a calculated binding free energy of −10.01 kcal/mol (Figure 8E). Finally, fucoxanthin formed hydrogen bonds with PHE−298 (two bonds: 1.8 Å, 2.5 Å), LYS−534 (1.9 Å), LEU−566 (1.9 Å), and GLU−533 (two bonds: 2.1 Å, 2.9 Å) on the TFR1 protein; the calculated binding free energy was −7.91 kcal/mol (Figure 8F). All binding free energies indicated high binding efficiency. Our molecular docking analyses revealed both hydrogen bonding and hydrophobic interactions between fucoxanthin and its target proteins. Detailed descriptions of these interactions are provided in the Supplementary Material (Tables S1–S12).

## 3. Discussion

Tongue squamous cell carcinoma is a unique subtype of head and neck squamous cell carcinomas with aggressive features and high rates of recurrence and metastasis [20]. Surgery is the main treatment, followed by adjuvant radiotherapy and chemotherapy; the 5−year overall survival rate is approximately 50% [21]. However, because of adverse reactions to chemotherapy drugs and limited treatment strategies, there is an urgent need to develop new therapeutic agents for tongue cancer [22]. Many natural products and their constituents have attracted increasing attention due to potent anti−tumor properties and low toxicity [23]. Fucoxanthin, a major carotenoid in brown algae, is a small molecule with unique chemical structure. Fucoxanthin’s pharmacological activities confer anti−tumor, anti−obesity, anti−angiogenic, anti−diabetic, anti−inflammatory, anti−oxidative, anti−aging, and protective properties [24]. In the present study, we found that SCC−25 cell viability decreased as the fucoxanthin concentration increased; the IC_50_ value for fucoxanthin in SCC−25 cells was 4.17 nM. Additional experiments showed that fucoxanthin strongly induced apoptosis and promoted ROS accumulation in SCC−25 cells.

Ferroptosis is a novel mode of cell death, first described by Dixon in 2012 [3]. In contrast to apoptosis, necrosis, autophagy, and other forms of death, ferroptosis is caused by iron−dependent lipid peroxidation. The core mechanism of ferroptosis involves lipid peroxidation and iron accumulation, cyclooxygenase−2 (COX2) upregulation, GSH depletion, and GPX4 inactivation [25]. GPX4, an antioxidant enzyme and key regulator of ferroptosis, uses GSH as a cofactor to catalyze lipid peroxide reduction. Simultaneously, GSH is affected by SLC7A11 [26]. The cellular iron regulator TFR1 is a transmembrane glycoprotein that transports iron and stores it in a non−toxic form within the ferritin metalloprotein complex [27]. Ferroptosis, triggered by ischemia and hypoxia occurring after cell injury, results from TFR1 dysfunction, iron homeostasis imbalance, excessive Fenton reaction induction, and generation of harmful ROS and lipid peroxidation products. TFR1 accumulation on the cell surface is an important ferroptosis indicator [28]. In the present study, reverse transcription–quantitative polymerase chain reaction (RT−qPCR) and Western blotting were used to assess the mRNA and protein expression patterns of TFR1, revealing that fucoxanthin promoted ferroptosis in SCC−25 cells by promoting TFR1 expression.

The transcription factor p53 plays a key role in tumor cell inhibition. In recent years, its involvement in ferroptosis has received increasing research interest, and there is evidence of a dual regulatory role [29]. Specifically, p53 can promote ferroptosis by downregulating SLC7A11 or upregulating spermidine/spermine N1−acetyltransferase 1 (SAT1) and glutaminase 2 (GLS2). It also can inhibit ferroptosis by suppressing dipeptidyl peptidase 4 (DPP4) activity or by upregulating cyclin−dependent kinase inhibitor 1A (CDKN1A/P21) [30]. This p53/SLC7A11 regulatory axis could sensitize SCC−25 cells to fucoxanthin−induced ferroptosis by increasing p53 expression in fucoxanthin−treated cells.

Keap1 is an important upstream negative regulator of Nrf2. Studies have shown that p62 can promote Nrf2 activation via Keap1, thereby inhibiting ferroptosis in hepatocellular carcinoma cells [31]. In the present study, fucoxanthin significantly inhibited SCC−25 cell activity in a concentration−dependent manner. Additionally, fucoxanthin significantly increased the level of apoptosis in SCC−25 cells. Further analyses showed that fucoxanthin downregulates Keap1 and Nrf2 expression at the gene and protein levels. Considering that fucoxanthin can inhibit ferroptosis, these findings suggest that the effect of fucoxanthin on ferroptosis is mediated by the Keap1/Nrf2 pathway. Nrf2 is a major regulator of antioxidant and detoxification processes. It can increase the expression levels of SLC7A11 and GPX4; it also promotes ferroptosis by binding to downstream antioxidant response element (ARE) genes [32]. Among these genes, the HO−1 promoter region is the most common binding site; HO−1 inhibition promotes ferroptosis through various mechanisms [33]. HO−1 is an important antioxidant enzyme that catalyzes the catabolism of heme into ferrous iron, carbon monoxide, and biliverdin [34]. Heme degradation helps to prevent pro−oxidation; the byproduct biliverdin and its reduced form, bilirubin, exhibit potent ROS scavenging activity [35]. HO−1 is an important source of cellular iron. During cell death triggered by the ferroptosis inducer erastin, HO−1 initiates ferroptosis via membrane lipid peroxidation. The Nrf2/HO−1 axis is a central regulator of cellular antioxidant responses; recent studies have shown that aberrant activation of the Nrf2/HO−1 axis frequently occurs in cancer cells and tumor tissues [36]. Nrf2 and HO−1 levels are elevated in various human malignancies; therefore, inhibition of the Nrf2/HO−1 pathway has been speculated to promote ferroptosis [37]. Consistent with this notion, we found that Nrf2 and HO−1 expression levels were reduced in fucoxanthin−treated cells. These results suggest that fucoxanthin inhibits activation of the Keap1/Nrf2/HO−1 pathway, leading to increased levels of lipid peroxidation and ROS formation, thereby promoting ferroptosis and disrupting redox homeostasis in tongue cancer cells. Additionally, molecular docking results showed that fucoxanthin could bind to all target proteins (p53, SLC7A11, GPX4, Keap1, HO−1, and TFR1). Fucoxanthin exhibited strong hydrogen bond interactions with these residues at short distances, suggesting stable complex formation. Overall, these findings indicate that fucoxanthin is a potential therapeutic agent for tongue cancer, highlighting the need for further investigation to facilitate clinical applications.

## 4. Materials and Methods

### 4.1. Cell Culture

SCC−25 human tongue cancer cells and 293T human embryonic kidney cells were obtained from the Shanghai Institute of Cell Biology, Chinese Academy of Sciences (Shanghai, China). The cells were cultured in Dulbecco’s modified Eagle medium (DMEM) supplemented with 10% fetal bovine serum and 1% penicillin/streptomycin in an incubator at 37 °C with 5% CO_2_. The medium was changed daily, and cells in the logarithmic growth phrase were used for experiments.

### 4.2. MTT Assay to Detect Cell Viability

SCC−25 tongue cancer cells and 293T embryonic kidney cells were seeded in 96−well plates at a density of 5 × 10^3^ cells per well. When cells reached 70–80% confluence, they were treated with fucoxanthin (provided by Zhejiang Wanli University; purity > 95% as determined by high−performance liquid chromatography) at concentrations of 0, 1, 2, 3, 4, 5, 6, 7, and 8 nM for 24 or 48 h. 3−(4,5−dimethylthiazol−2−yl)−2,5−diphenyltetrazolium bromide (MTT; final concentration 0.5 mg/mL, Solarbio, Beijing, China) was added to each well, and the cells were incubated at 37 °C for 4 h. The medium was then removed and dimethyl sulfoxide was added to dissolve the formazan in the cells, and the plates were shaken for 10 min using a shaking table. Finally, absorbance values were measured at 570 nm.

### 4.3. ROS Detection by Flow Cytometry

SCC−25 cells in the logarithmic growth phase were seeded in 6−well plates at a density of 2 × 10^5^ cells/mL. When cells reached 70–80% confluence, they were treated with 0, 2, 4, or 6 nM fucoxanthin and cultured for 48 h. The medium was then removed and the probe dichlorodihydrofluorescein diacetate (DCFHDA, diluted in serum−free medium; Solarbio) was added. The cells were incubated at 37 °C for 20 min, then detached from the plates and analyzed via flow cytometry (BD FACSVerse, Franklin Lakes, NJ, USA). The flow cytometry data were analyzed by FlowJo_V10 software (Tree Star, Ashland, OR, USA).

### 4.4. Measurement of MDA, GSH, SOD, and Fe Levels

SCC−25 cells in the logarithmic growth phase were seeded in 6−well plates at a density of 2 × 10^5^ cells/mL. When cells reached 70–80% confluence, they were treated with 0, 2, 4, or 6 nM fucoxanthin and cultured for 48 h. The cells were collected by trypsin digestion and sonicated in an ice bath. Subsequently, the cells were processed in accordance with the instructions of the MDA, GSH, SOD, and Fe kits (Solarbio, Beijing, China). Absorbances were measured at 532 nm, 412 nm, 450 nm, and 510 nm using a microplate reader (Molecular Devices, San Jose, CA, USA).

### 4.5. Apoptosis Detection by Flow Cytometry

SCC−25 cells in the logarithmic growth phase were seeded in 6−well plates at a density of 2 × 10^5^ cells/mL. When cells reached 70–80% confluence, they were treated with 0, 2, 4, or 6 nM fucoxanthin and cultured for 48 h. The cells were collected by trypsin digestion, suspended in 1× binding buffer, and stained with 5 μL Annexin V–fluorescein isothiocyanate (FITC) dye and PI staining solution (BD, Tokyo, Japan) for 15 min in the dark. Apoptotic cells were detected by flow cytometry, and the apoptosis rate for each treatment group was calculated.

### 4.6. Mitochondrial Membrane Potential Measurement by Flow Cytometry

SCC−25 cells in the logarithmic growth phase were seeded in 6−well plates at a density of 2 × 10^5^ cells/mL. When cells reached 70–80% confluence, they were treated with 0, 2, 4, or 6 nM fucoxanthin and cultured for 48 h. The cells were collected by trypsin digestion and incubated with JC−1 staining solution (BD, Tokyo, Japan) in the dark. After incubation, cells were washed with buffer and analyzed.

### 4.7. RT−qPCR Analysis of Ferroptosis−Related Gene Expression

Total RNA was extracted in accordance with the instructions of the RNA extraction kit (Magen, Guangzhou, China); its concentration and purity were then determined. A260/A280 values for all RNA samples were between 1.8 and 2.0. First−strand cDNA was synthesized according to the instructions provided with the reverse transcription kit (Tran, Beijing, China) and used as a template for RT−qPCR (20 μL reaction volume). The amplification conditions were pre−denaturation at 94 °C for 1 min, followed by 40 cycles of denaturation at 94 °C for 5 s, annealing at 50–60 °C for 15 s, and extension at 72 °C for 10 s. Relative mRNA expression levels of p53, SLC7A11, GPX4, Nrf2, Keap1, HO−1, and TFR1 were calculated using the 2^−ΔΔCt^ method, with β−actin as the internal control. The primer sequences are listed in Table 1.

### 4.8. Western Blotting Analysis of Ferroptosis−Related Protein Expression

Cells were seeded in 6−well plates (2 × 10^5^ cells/well) and treated with 2, 4, or 6 nM fucoxanthin for 48 h. Cell lysis buffer containing protease and phosphatase inhibitors was added to cultures and cells were lysed on ice for 30 min, then centrifuged at 15,000× *g* for 15 min at 4 °C; the resulting supernatant was collected. After protein concentrations had been determined using the bicinchoninic acid (BCA) method, supernatants were mixed with 5× loading buffer for denaturation. Thirty micrograms of protein per sample were separated by 10% sodium dodecyl sulfate–polyacrylamide gel electrophoresis and electrotransferred to polyvinylidene fluoride (PVDF) membranes. The membranes were blocked with 5% skim milk powder for 1 h and incubated with primary antibodies (all 1:1000; see below) overnight at 4 °C. After membranes had been washed three times with Tris−buffered saline plus Tween (TBST), they were incubated with secondary antibodies (all 1:500) for 1 h at room temperature. After additional washes with TBST, enhanced chemiluminescence reagent was added and bands were detected using an imaging device (Tanon, Shanghai, China). Relative protein expression levels of SLC7A11, GPX4, TFR1, HO−1, Keap1, Nrf2, and p53 (all antibodies from ABclonal Technology, Wuhan, China) were calculated using ImageJ 1.53 software, with glyceraldehyde−3−phosphate dehydrogenase (GAPDH) as the internal control.

### 4.9. Molecular Docking

The chemical structure of fucoxanthin was depicted using ChemDraw 19.0 software and subjected to energy minimization within Chem3D 14.0 software. The following protein structures were retrieved from the Protein Data Bank (https://www.rcsb.org/, (accessed on 4 March 2024)) and saved in pdb format: p53 (PDB ID: 3KMD), SLC7A11 (PDB ID: 7CCS), GPX4 (PDB ID: 5H5Q), Keap1 (PDB ID: 6QMC), HO−1 (PDB ID: 6EHA), and TFR1 (PDB ID: 7ZQS). PyMOL 2.5.2 was used to remove water molecules and eliminate modified ligands. AutoDock Tools 1.5.7 (https://ccsb.scripps.edu/mgltools/, (accessed on 13 May 2024) La Jolla, CA, USA) was used to convert fucoxanthin and SLC7A11, GPX4, TFR1, HO−1, Keap1, Nrf2, and p53 proteins into pdbqt format for molecular docking. LigPlus 2.5.5 was used to analyze hydrogen bonding, hydrophobic interactions, and other forces between ligands and target proteins.

### 4.10. Data Analysis

GraphPad Prism8.3 software was used for all statistical analyses. Each experiment was repeated three times, and all data are expressed as means ± SDs. Differences between groups in each experiment were analyzed by one−way analysis of variance (ANOVA). The statistical significance threshold was defined as *p* < 0.05.

## 5. Conclusions

Fucoxanthin inhibits the Nrf2−HO−1/SLC7A11 pathway and disrupts anti−oxidative stress defenses in SCC−25 cells, causing imbalances in iron and GSH metabolism. Excess ferrous iron induces the Fenton reaction and GSH depletion inactivates GPX4; HO−1 inhibition blocks ROS removal. Finally, this process leads to ROS accumulation and ferroptosis onset. The present study showed that fucoxanthin exhibits good anti−tumor activity in SCC−25 cells, providing an experimental basis for its use in the treatment of tongue cancer. These findings should be validated by in vivo experiments.

## Figures and Tables

**Table 1:** Primers used for RT-qPCR.

Gene	Sense Primer (5′-3′)	Antisense Primer (3′-5′)
*p53*	GCGTGTGGAGTATTTGGATGAC	AGTGTGATGATGGTGAGGATGG
*TFR1*	AACTCAGCAAAGTCTGGCGT	GACCCCCAATACACCGCATA
*SLC7A11*	TCCTGCTTTGGCTCCAT	ACAGGCGTTCGTGTGAGGAGA
*GPX4*	ACAAGAACGGCTGCGTGGTGAA	AGATCGAGGTGTTCACACACCG
*Nrf2*	TACTCCCAGGTTGCCCACA	AAGGGCAAACATCTAC
*Keap1*	GTGTCCATTGAGGGTATCCACC	GCTCAGCGAAGTTGGCGAT
*HO-1*	GGCCTCCCTGTACCACATCT	GGATGTGTGGTCGGTACGTC
*β-actin*	CCTGGCACCCAGCACAAT	GGGCCGGACTCGTCATAC

## Data Availability

The data presented in this study are available on request from the corresponding author.

## Supplementary Materials

The following supporting information can be downloaded at: https://www.mdpi.com/article/10.3390/molecules29122832/s1, Table S1: Hydrophobic interaction between fucoxanthin and p53; Table S2: Hydrogen bond interactions be-tween fucoxanthin and p53; Table S3: Hydrophobic interaction between fucoxanthin and SLC7A11; Table S4: Hydrogen bond interactions between fucoxanthin and SLC7A11; Table S5: Hydrophobic interaction between fucoxanthin and GPX4; Table S6: Hydrogen bond interactions between fuco-xanthin and GPX4; Table S7: Hydrophobic interaction between fucoxanthin and Nrf2-Keap 1; Table S8: Hydrogen bond interactions between fucoxanthin and Nrf2-Keap 1; Table S9: Hydrophobic in-teraction between fucoxanthin and HO-1; Table S10: Hydrogen bond interactions between fuco-xanthin and HO-1; Table S11: Hydrophobic interaction between fucoxanthin and TFR1; Table S12: Hydrogen bond interactions between fucoxanthin and TFR1.
