# Supplementary material for: Fucoxanthin Induces Ferroptosis in Cancer Cells via Downregulation of the Nrf2/HO−1/GPX4 Pathway
Source: Molecules. 2024 Jun 14;29(12):2832. doi: 10.3390/molecules29122832 (PMC11206433; doi:10.3390/molecules29122832)
Supplement: Supplementary file 1 [file molecules-29-02832-s001.zip › molecules-3033580-supplementary.pdf]

Supplementary Materials:

1. Interaction between fucoxanthin and p53

Table S1. Hydrophobic interaction between fucoxanthin and p53.

Residue	AA	Distance	Ligand Atom	Protein Atom
100A	GLN	3.78	1998	72
111A	LEU	3.41	1983	194
113A	PHE	3.34	1955	211
113A	PHE	3.43	1958	209
126A	TYR	3.97	1957	327
126A	TYR	3.46	1965	325
128A	PRO	3.13	1971	343
128A	PRO	3.31	1965	344

Table S2. Hydrogen bond interactions between fucoxanthin and p53.

Residue	AA	Distance H-A	Distance D-A	Donor Angle	Protein donor?	Side chine	Donor Atom	Acceptor Atom
102A	THR	1.87	2.85	161.05	√	×	91 [Nam]	1999 [O3]

2. Interaction between fucoxanthin and SLC7A11

Table S3. Hydrophobic interaction between fucoxanthin and SLC7A11.

Residue	AA	Distance	Ligand Atom	Protein Atom
103B	THR	3.29	4204	489
104B	ILE	3.86	4200	500
104B	ILE	3.22	4203	498
117B	THR	3.15	4199	616
118B	PHE	3.68	4236	626
409B	TYR	3.45	4231	3299
409B	TYR	3.14	4227	3301
410B	LEU	3.65	4198	3313
414B	LYS	3.85	4225	3365
414B	LYS	3.97	4218	3363
416B	ASP	3.78	4216	3383
417B	LEU	4.00	4204	3395

Table S4. Hydrogen bond interactions between fucoxanthin and SLC7A11.

Residue	AA	Distance H-A	Distance D-A	Donor Angle	Protein donor?	Side chine	Donor Atom	Acceptor Atom
106B	LYS	2.12	3.02	146.44	√	√	517 [N3+]	4210 [O2]
415B	PRO	2.22	2.95	132.20	×	×	4222 [O3]	3374 [O2]

3. Interaction between fucoxanthin and GPX4

Table S5. Hydrophobic interaction between fucoxanthin and GPX4.

Residue	AA	Distance	Ligand Atom	Protein Atom
132A	LYS	3.64	1858	997
133A	ILE	3.18	1846	1010
141A	HIS	3.61	1846	1077
142A	PRO	3.95	1824	1090
908B	TRP	3.84	1840	1742

Table S6. Hydrogen bond interactions between fucoxanthin and GPX4.

Residue	AA	Distance H-A	Distance D-A	Donor Angle	Protein donor?	Side chine	Donor Atom	Acceptor Atom
34A	ASP	2.23	3.13	155.63	×	√	1842 [O3]	20 [O2]
36A	ARG	2.07	3.04	158.26	√	√	45 [Ng+]	1842 [O3]
111A	GLY	2.40	3.06	125.44	×	×	1864 [O3]	795 [O2]

4. Interaction between fucoxanthin and Nrf2-Keap 1

Table S7. Hydrophobic interaction between fucoxanthin and Nrf2-Keap 1.

Residue	AA	Distance	Ligand Atom	Protein Atom
420A	VAL	3.46	2756	888
467A	VAL	3.11	2764	1340
607A	ALA	3.36	2752	2695

Table S8. Hydrogen bond interactions between fucoxanthin and Nrf2-Keap 1.

Residue	AA	Distance H-A	Distance D-A	Donor Angle	Protein donor?	Side chine	Donor Atom	Acceptor Atom
416A	ILE	2.20	3.13	162.55	×	×	2790 [O3]	860 [O2]
467A	VAL	2.02	2.91	143.80	√	×	1333 [Nam]	2769 [O3]
512A	VAL	2.30	3.60	142.79	×	×	2770 [O3]	1323 [O2]

5. Interaction between fucoxanthin and HO-1

Table S9. Hydrophobic interaction between fucoxanthin and HO-1.

Residue	AA	Distance	Ligand Atom	Protein Atom
37A	PHE	3.53	2161	279
50A	VAL	3.74	2159	413
54A	LEU	3.63	2157	446
140A	ASP	3.44	2145	1329
147A	LEU	3.42	2152	1387
147A	LEU	3.42	2161	1385
166A	PHE	3.46	2146	1556
167A	PHE	3.11	2146	1565
167A	PHE	3.87	2161	1567
169A	PHE	3.03	2167	1584
169A	PHE	3.01	2139	1586
169A	PHE	3.10	2142	1588
175A	ALA	3.47	2169	1637
214A	PHE	3.58	2156	2047
214A	PHE	3.48	2155	2045

Table S10. Hydrogen bond interactions between fucoxanthin and HO-1.

Residue	AA	Distance H-A	Distance D-A	Donor Angle	Protein donor?	Side chine	Donor Atom	Acceptor Atom
173A	ALA	2.27	3.02	133.89	×	×	2183 [O3]	1622 [O2]

6. Interaction between fucoxanthin and TFR1

Table S11. Hydrophobic interaction between fucoxanthin and TFR1.

Residue	AA	Distance	Ligand Atom	Protein Atom
259B	ALA	3.12	6123	1342
263B	THR	3.20	6109	1376
264B	PHE	3.33	6114	1388
264B	PHE	3.73	6121	1390
288B	PHE	3.59	6123	1612
288B	PHE	3.55	6124	1611
297B	PHE	3.36	6121	1689
533B	GLU	3.58	6087	3879

Table S12. Hydrogen bond interactions between fucoxanthin and TFR1.

Residue	AA	Distance H-A	Distance D-A	Donor Angle	Protein donor?	Side chine	Donor Atom	Acceptor Atom
298B	PHE	2.51	3.25	129.26	√	×	1693 [Nam]	6127 [O3]
298B	PHE	1.82	2.72	154.63	×	×	6127 [O3]	1697 [O2]
533B	GLU	2.90	2.91	105.21	√	√	3882 [O3]	6093 [O2]
534B	LYS	2.08	3.09	170.39	√	×	3884 [Nam]	6093 [O2]
534B	LYS	1.94	2.96	172.14	√	√	3893 [N3+]	6105 [O3]
566B	LEU	1.91	2.85	163.20	×	×	6125 [O3]	4178 [O2]
